# Supplementary material for: Jamming and unusual charge density fluctuations of strange metals
Source: Nat Commun. 2023 Jul 3;14:3919. doi: 10.1038/s41467-023-39499-x (PMC10318073; doi:10.1038/s41467-023-39499-x)
Supplement: Supplementary file 1 — Supplementary Information [file 41467_2023_39499_MOESM1_ESM.pdf]

Supplementary Information

Stephen J. Thornton,[1] Danilo B. Liarte,[1, 2, 3] Peter Abbamonte,[4] James P. Sethna,[1] and Debanjan Chowdhury[1, *]

[1] *Department of Physics, Cornell University, Ithaca NY 14853, U.S.A.*
[2] *ICTP South American Institute for Fundamental Research, São Paulo, SP, Brazil*
[3] *Institute of Theoretical Physics, São Paulo State University, São Paulo, SP, Brazil*
[4] *Department of Physics, University of Illinois at Urbana-Champaign, Urbana IL 61801, U.S.A.*

(Dated: June 5, 2023)

I. EFFECTIVE MEDIUM THEORY

As noted in the main text, we model the system as a layered structure consisting of two-dimensional randomly populated lattices of harmonic springs. The local deformations associated with the configurations (e.g. compression) are assumed to capture the fluctuations associated with an underlying electronic liquid; see Supplementary Fig. 1. We make an analogy between the strongly overdamped plasmon in a class of strange metals and the strongly overdamped phonon in viscoelastic systems near the onset of rigidity. One of the features of rigidity percolation (and jamming) is a nearly flat density of states at the transition, which corresponds to an anomalously large number of low-energy excitations into which an excited phonon can decay. This is captured in randomly populated networks analytically by an effective-medium theory known as the coherent potential approximation (CPA) [5].

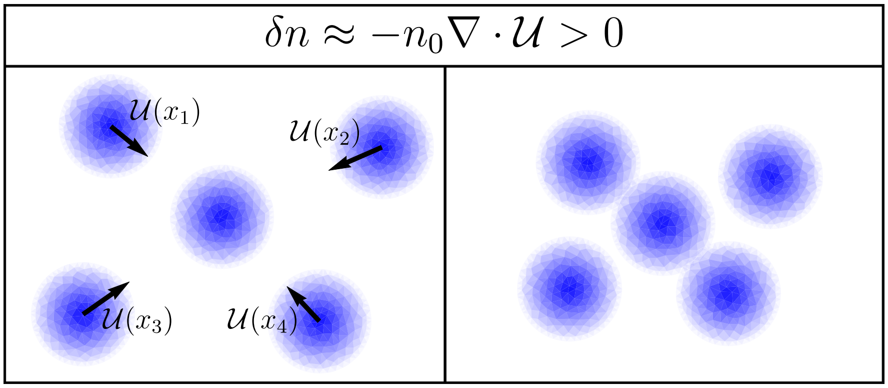

SUPPLEMENTARY FIG. 1. The density-density response is connected to longitudinal fluctuations of the displacement. Shown here is a local compression wave increasing the local density of charged particles; the precise composition of the charged low-energy excitations is not known.

In this model, we begin with a fully occupied lattice with unit elastic coefficients (harmonic springs) and randomly dilute the lattice, keeping each bond with probability p. To study the dynamics of the disordered system, the CPA self-consistently replaces the Green's function of this randomly populated lattice with a spatially homogeneous disorder-averaged Green's function. The self-energy calculated in this framework is typically recast as a frequency-dependent elastic coefficient $\mathcal{K}(\omega)$. More generally, given several bond occupation probabilities $\{p_\alpha\}$, the CPA gives frequency-dependent elastic coefficients for each sublattice $\{\mathcal{K}_\alpha(\omega)\}$, where $\alpha = 1, 2 \ldots$ is the sublattice index. This method faithfully reproduces the zero-frequency athermal phase diagram and phase transitions predicted by Maxwell's constraint counting arguments, where the number of constraints is equal to the number of degrees of freedom [5]. There is also experimental evidence that it properly reproduces the low-frequency behavior of other microscopically disordered systems undergoing rigidity transitions, such as soft gels [1, 2].

A specific type of phase transition that these randomly populated lattices can undergo is broadly known as *rigidity percolation*, when a disordered elastic network loses rigidity in a continuous fashion at $p = p_c$. This can be seen

* debanjanchowdhury@cornell.edu

by examining the effective bulk modulus B and the shear modulus G of a bond-diluted lattice, defined by how the potential energy of the lattice U changes in response to small linearized strains u_{ij}:

$$\Delta U = \frac{B}{2}\left(u_{xx}+u_{yy}\right)^2 + 2G\left[u_{xy}^2 + \frac{1}{4}\left(u_{xx}-u_{yy}\right)^2\right]. \tag{1}$$

The predicted zero-frequency bulk and shear moduli of these diluted lattices grow continuously from 0 across such a transition, as p grows larger than p_c. The density-density response near jamming is discontinuous across the critical point due to the jump in the bulk modulus, as opposed to the response near RP. The MEELS phenomenology is better captured in terms of RP, even though the nature of the anomalous low-energy excitations in the vibrational density of states is the same for both. This paper focuses on the *universal* behavior of *rigidity percolation*, that is, the characteristics of the phase transition that do not depend on the underlying microscopic lattice geometry.

For a single sublattice in two dimensions, the self-consistent equation for the elastic coefficients reads

$$\frac{p-\mathcal{K}(\omega)}{1-\mathcal{K}(\omega)} = \frac{1}{z}\frac{1}{s_{\mathrm{BZ}}}\int_{\mathrm{BZ}} \mathrm{d}^2 q\ \mathrm{Tr}\left[D\left(q,\mathcal{K}(\omega)\right)\left(D\left(q,\mathcal{K}(\omega)\right)-\omega^2 I\right)^{-1}\right] \tag{2}$$

where p is as defined earlier, $D(...)$ is the dynamical matrix, and z is the average number of bonds per unit cell. The derivation of the more general form of this expression can be found, for instance, in the supplemental material of [5].

By expanding the dynamical matrix for a general 2D isotropic lattice in the long-wavelength limit as

$$D_{ij}\left(\mathcal{K}(\omega)\right) = B\left(\mathcal{K}(\omega)\right) q^2 \hat{q}_i \hat{q}_j + G\left(\mathcal{K}(\omega)\right) q^2 \delta_{ij}, \tag{3}$$

we can evaluate the integral and solve the self-consistency equations. The combination $K_{\mathrm{L}} \equiv B+G$ is the only combination of moduli that enters the longitudinal response (and the density-density response), and must be proportional to the only microscopic coupling in the problem $\mathcal{K}$. Writing the self-consistent equation for K_{L} and expanding to linear order in the distance to the continuous critical point $\delta p \equiv p - p_c$, one finds an expression involving only certain invariant scaling combinations. The coupling is found as the self-consistent solution to

$$\pm 1 - \widetilde{K}_{\mathrm{L}\pm} = \frac{\widetilde{\omega}'^2}{\widetilde{K}_{\mathrm{L}\pm}} \log\left(-\frac{\widetilde{K}_{\mathrm{L}\pm}}{\widetilde{\omega}'^2 \widetilde{Z}}\right), \tag{4a}$$

$$\widetilde{K}_{\mathrm{L}\pm} \equiv \frac{K_{\mathrm{L}\pm}}{K_{\mathrm{L}0}\left|\delta p\right|}, \tag{4b}$$

$$\widetilde{\omega}' \equiv \frac{\omega}{\omega_0'\left|\delta p\right|}, \tag{4c}$$

$$\widetilde{Z} \equiv Z_0 \left|\delta p\right|. \tag{4d}$$

Here $K_{\mathrm{L}0}$, ω_0', and Z_0 are non-universal constants of order unity, and we choose their values from the bond-diluted mechanical triangular lattice with nearest-neighbor bonds for all plots. The $+$ and $-$ in the self-consistent equations refer to the "solid" ($p > p_c$) and "floppy" ($p < p_c$) sides of the transition, respectively. Asymptotically close to the transition, the solution to the above self-consistent effective medium equation is given by

$$\widetilde{K}'_{\mathrm{L}\pm}\left(\widetilde{\omega}''\right) = \pm 1 + \sqrt{1-\widetilde{\omega}''^2}, \tag{5}$$

with $\widetilde{K}'_{\mathrm{L}\pm} \equiv K_{\mathrm{L}\pm}/K''_{\mathrm{L}0}\left|\delta p\right|$ and $\widetilde{\omega}'' \equiv \omega/\omega_0''\left(\left|\delta p\right| / \left|\log\left(\left|\delta p\right|\right)\right|^{1/2}\right)$. The logarithm that appears in this scaling variable for the frequency is unique to 2 dimensions and does not affect the qualitative results of any of our calculations; this will be elaborated upon in a future manuscript. The imaginary part of the square root should be interpreted as non-positive for causality reasons. The dissipation that is calculated within the CPA framework aims to capture phonon scattering off of "defects" introduced by the disorder. The vanishing of the imaginary part of $K_{\mathrm{L}}(\omega)$ at small but finite frequency does not survive universal corrections to scaling; when the full CPA self-consistency equation is solved on the solid side ($\delta p > 0$), then $\mathrm{Im}\left(K_{\mathrm{L}}\right) < 0$ for all $\omega > 0$.

II. UNIVERSAL SCALING OF THE DENSITY-DENSITY RESPONSE

The frequency-dependent elastic coefficient derived in the previous section can be used to determine the effective bulk and shear moduli of the elastic medium, which can be used to determine the long-wavelength density-density response. The form of our long-wavelength density-density response is calculated as follows: the equation of motion for an isotropic elastic sheet without external damping reads

$$\rho \ddot{\mathcal{U}} = B\nabla\left(\nabla\cdot\mathcal{U}\right) + G\nabla^2\mathcal{U} + \mathbf{f}^{\mathrm{ext}} \tag{6}$$

where ρ is the (constant) average background density and $\mathcal{U}$ is a small displacement field. B and G are proportional to $\mathcal{K}(\omega)$, and are hence frequency-dependent and complex. Assuming the local perturbation due to the external forcing field to be small, we expand $n \approx n_0 (1 - \nabla \cdot \mathcal{U}) = \rho/m (1 - \nabla \cdot \mathcal{U})$; see Supplementary Fig. 1.

We then use the definition of the susceptibility as the change in the density as a result of the perturbing conjugate field, $\Pi \equiv -\delta n/\delta h$. Note that the additional negative sign in front of the susceptibility compared to [6, 7] is related to differing definitions for the susceptibility; we adopt the experimentalists' convention [8] where the imaginary part is negative for positive frequencies. Just as $\mathcal{U}$ and f are thermodynamic conjugates (because they enter the energy density as $\mathcal{U} \cdot f$), so are $\delta n_q = i n_0 q \cdot \mathcal{U}_q$ and $\delta h_q = f_q^{\mathrm{Lext}}/i n_0 q$. Thus,

$$\left(-\rho\omega^2 + (B+G)\,q^2\right)\delta n_q = n_0^2 q^2 \delta h_q, \tag{7}$$

$$\Pi \equiv -\frac{\delta n_q}{\delta h_q} = \frac{\rho^2 q^2}{m^2}\frac{1}{\rho\omega^2 - K_L(\omega)\,q^2}. \tag{8}$$

This allows us to write a universal form for the density-density response at rigidity percolation on both sides of the transition:

$$\widetilde{\Pi} = \frac{\widetilde{q}^2}{\widetilde{\omega}''^2 - \left(\sqrt{1-\widetilde{\omega}''^2} \pm 1\right)\widetilde{q}^2} \tag{9}$$

where the appropriate scaling is found to be $\widetilde{q} \equiv q/q_0 \left(|\delta p|^{1/2} / |\log(|\delta p|)|^{1/2}\right)$ and $\widetilde{\Pi} \equiv \Pi\,|\delta p|\,/\Pi_0$. The $O(1)$ constants q_0 and Π_0 are non-universal. Since the scaling of B, G, and ω near rigidity percolation has already been fixed by the self-consistency equation, the scaling of q is found by balancing powers of δp in the denominator of the expression for Π. In practice, the corrections to scaling that fix the imaginary part at low frequencies are significant enough (they vanish as $\sim |\log|\delta p||^{-1/2}$) that we numerically solve the self-consistent equations with $\widetilde{Z}$ included to find a more faithful representation of the CPA predictions. Finally, the effects of the long-ranged Coulomb interaction are added using the RPA (as described in the main text):

$$\chi(q,\omega) = \frac{\Pi(q,\omega)}{1 - V(q)\Pi(q,\omega)} \tag{10}$$

with the 3D Coulomb interaction $V(q) = 4\pi e^2/q^2$.

The qualitative features that the CPA predicts for χ reflect what is expected of a lattice near a rigidity transition: sharply defined quasiparticles exist only at the longest wavelengths, and rapidly broaden with increasing q into an incoherent bump at a frequency $\Delta\omega_\star$ set by the distance to the rigidity percolation transition. If we are near the critical point, experimental probes of the response will inevitably probe only the region of large $\widetilde{q}$, which leads to a q-independent shape of the response. In this model, a coherent quasiparticle can still be found near the center of the BZ.

For large values of $\widetilde{q}$, there is a range of frequencies where $\chi'' \sim \omega^{-1}$ decays slowly. For an experimental probe, this may indicate the violation of f-sum rules. However, at frequencies large enough, χ'' eventually decays as $\sim \omega^{-3}$ as predicted by Drude theory. Although the paradigm of proximity to a critical point with a large number of anomalously low-frequency modes serves to explain the q-independent shape of the response outside of the very center of the BZ, the shapes of the universal forms of the electronic response do not have the plateaus measured by the experiment. Because we have control over how our response depends on the distance to the critical point, we can investigate how long-wavelength disorder in a sample, represented by a distribution of distances to the critical point δp, modifies the observed form of the density-density response.

III. AVERAGING OVER THE LONG-WAVELENGTH SAMPLE DISORDER

We imagine an experimental sample prepared on average close to a critical point δp, where different regions of the sample are allowed to have slightly different distances to the critical point with spread σ. The effect of this long-wavelength disorder is then represented by averaging the response over many distances to the critical point using a Gaussian of center δp and width σ:

$$\overline{\chi_{\delta p}(q,\omega)} = \int_{-\infty}^{\infty} d\left(\Delta p'\right) P_\sigma(\Delta p')\ \chi_{\delta p'}(q,\omega) \tag{11}$$

$$P_\sigma[\Delta p'] = \frac{1}{\sqrt{2\pi\sigma^2}} e^{-\left(\Delta p'\right)^2/2\sigma^2}, \tag{12}$$

where $\chi_{\delta p}(q,\omega)$ is the response at a fixed distance (δp) from RP, and we choose a Gaussian distribution, $P_\sigma[\Delta p]$, with width σ and $\Delta p' \equiv \delta p' - \delta p$. One could imagine other methods for averaging over the effect of the long-wavelength disorder. In this case, we adopt the procedure where the susceptibility (related to an inverse stiffness at zero frequency) is estimated as an arithmetic mean of the susceptibilities of different portions of the sample; this means that a large susceptibility in any portion of the sample corresponds to a large average susceptibility. One could also estimate the susceptibility as the inverse of the average inverse susceptibility, which would suppress the susceptibility if there is a small susceptibility in any portion of the sample. For an inhomogeneous medium, the effective susceptibility is bounded between these two options [4].

The solution to the full self-consistency equations including the corrections to scaling cannot be written in terms of elementary functions, so we estimate the convolution by picking many values of δp, computing χ'' over a range of frequencies by solving the full self-consistency equations, and performing an appropriate weighted average of the χ'' at the experimental values of q (which correspond to asymptotically large $\widetilde{q}$ for windows of δp very close to the critical point). These averaged responses $\overline{\chi}''$ are compared with the measured imaginary part of the density-density response from the experiment. We seek to describe the universal behaviour of the electronic response, and so we will not capture the additional *lattice* phonon seen in the experimental data at the lowest frequencies in [3]. The plots in Supplementary Fig. 2 are generated near rigidity percolation using a width of $\sigma = 1.2 \times 10^{-3}$ in probability space. The central distances to RP chosen are $\delta p = 1.0 \times 10^{-3}$ and $\delta p = 1.4 \times 10^{-3}$ and exhibit q-independence over a selected range of frequencies for $q \gg |\delta p|^{1/2} / |\log(|\delta p|)|^{1/2}$.

Without disorder averaging, for $q \gg |\delta p|^{1/2} / |\log(|\delta p|)|^{1/2}$, the susceptibility has a broad bump near $\omega_\star$ associated with the decay of the quasiparticle into the anomalous low-frequency modes. When disorder averaging is performed, a sequence of these bumps leads to the emergence of a low-frequency plateau terminating near $\omega_\star$. Above the plateau, this theory predicts a region where $\chi'' \sim \omega^{-1}$ crossing over into a region where $\chi'' \sim \omega^{-3}$ (the high-frequency Drude scaling). In the experiment, an exponent $1 \le \alpha \le 3$ is measured out to the highest frequencies.

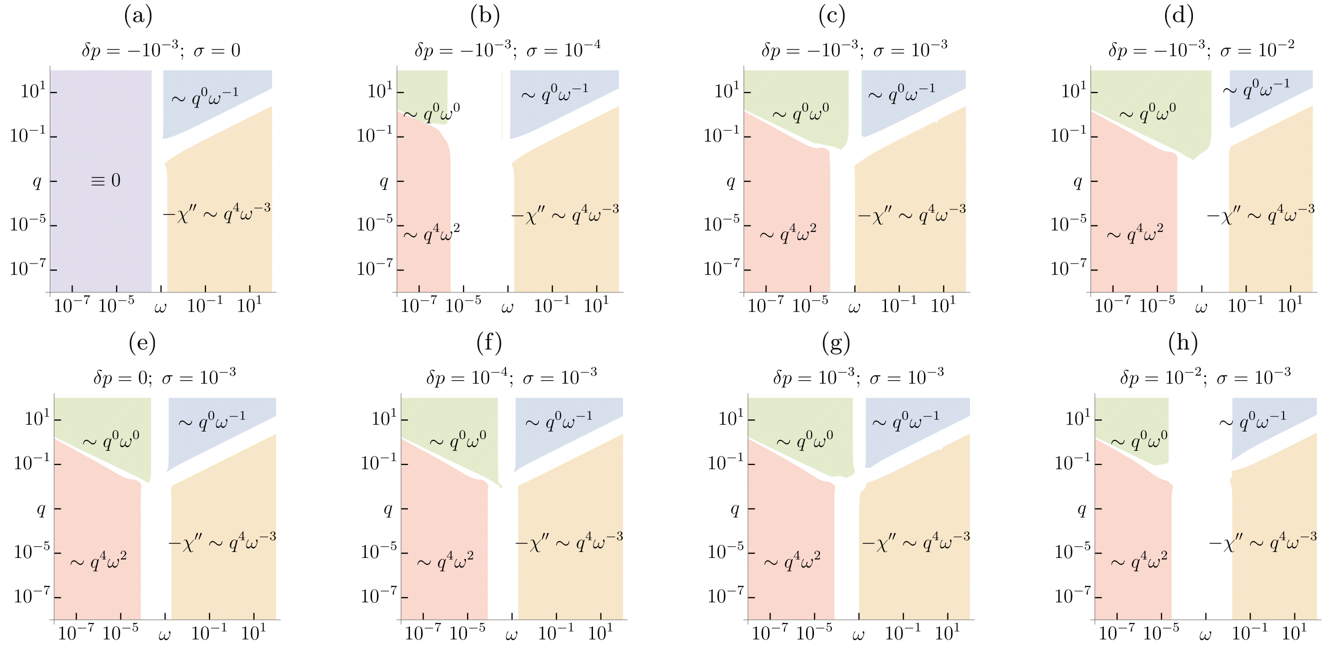

SUPPLEMENTARY FIG. 2. Scaling regimes of the disorder-averaged susceptibilities near the rigidity transition. Plots (a)–(d) show fixed $\delta p = -10^{-3}$ near the transition on the liquid side with increasing σ, demonstrating the emergence of the disorder-averaged plateau. Plots (e)–(h) show the effect of varying δp into the solid side at fixed $\sigma = 10^{-3}$. In this case, the disorder-averaging becomes less relevant and the peak shifts into higher frequencies with the predicted scaling. The region of exactly 0 imaginary part in (a) is eliminated by corrections to scaling. Plots are similar at large q on the solid and liquid sides.

The imaginary part of the susceptibility χ'' is odd in ω but contains a plateau that extends down to the lowest frequencies with increasing q. This results in a sharp feature at the lowest frequencies where the plateau ends and the response crosses over into power-law behavior to ensure $\chi''(q,0) = 0$. The location of this feature can be deduced from Supplementary Fig. 2, where the green ($-\chi'' \sim q^0\omega^0$) and red ($-\chi'' \sim q^4\omega^2$) regions touch. Plots of the lowest

frequency behavior are shown below in Supplementary Fig. 3 for the sake of completeness. In the experiment, the low frequency behavior shows a prominent feature tied to the lattice phonons, which is not included in our present theory.

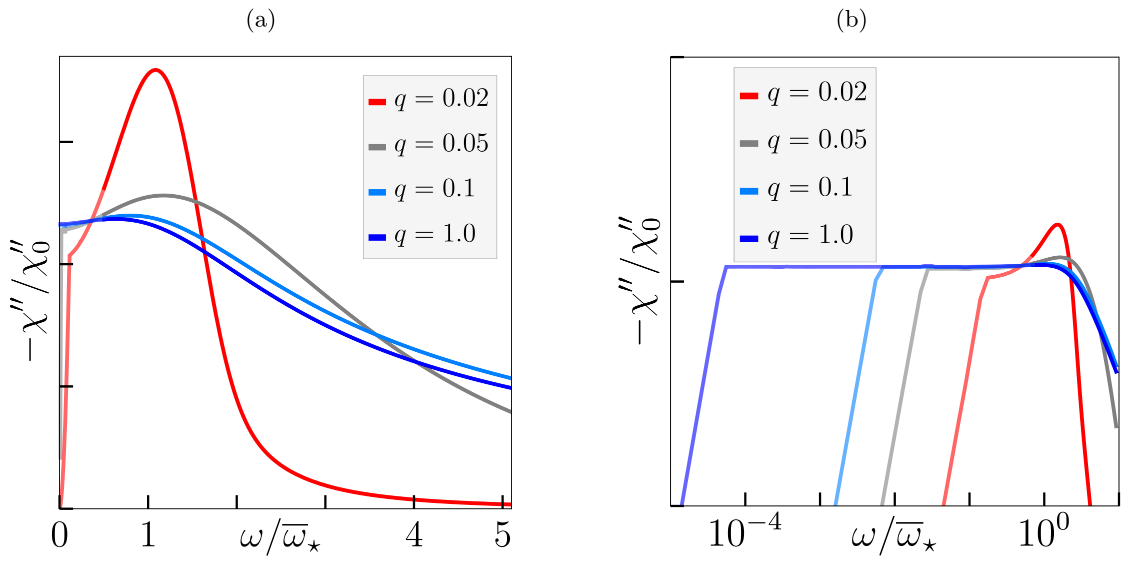

SUPPLEMENTARY FIG. 3. Low frequency behavior of the disorder-averaged response, including an anomalous "sharp" feature at the lowest frequencies where the plateau crosses over into a power-law regime enforcing $\chi''(q, 0) = 0$. (a) is shown on a linear-linear scale, while (b) is shown on a log-log scale.

[1] M. Bouzid, B. Keshavarz, M. Geri, T. Divoux, E. Del Gado, and G. H. McKinley. Computing the linear viscoelastic properties of soft gels using an optimally windowed chirp protocol. Journal of Rheology, 62(4):1037–1050, 2018.

[2] J. Colombo and E. Del Gado. Stress localization, stiffening, and yielding in a model colloidal gel. Journal of Rheology, 58(5):1089–1116, 2014.

[3] A. A. Husain, M. Mitrano, M. S. Rak, S. Rubeck, B. Uchoa, K. March, C. Dwyer, J. Schneeloch, R. Zhong, G. D. Gu, and P. Abbamonte. Crossover of charge fluctuations across the strange metal phase diagram. Phys. Rev. X, 9:041062, Dec 2019.

[4] R. V. Kohn and G. W. Milton. On bounding the effective conductivity of anisotropic composites. In J. L. Ericksen, D. Kinderlehrer, R. Kohn, and J.-L. Lions, editors, Homogenization and Effective Moduli of Materials and Media, pages 97–125, New York, NY, 1986. Springer New York.

[5] D. B. Liarte, X. Mao, O. Stenull, and T. Lubensky. Jamming as a multicritical point. Physical Review Letters, 122(12), Mar 2019.

[6] D. B. Liarte, S. J. Thornton, E. Schwen, I. Cohen, D. Chowdhury, and J. P. Sethna. Universal scaling for disordered viscoelastic matter II: Collapses, global behavior and spatio-temporal properties. arXiv e-prints, page arXiv:2202.13933, Feb. 2022.

[7] D. B. Liarte, S. J. Thornton, E. Schwen, I. Cohen, D. Chowdhury, and J. P. Sethna. Universal scaling for disordered viscoelastic matter near the onset of rigidity. Phys. Rev. E, 106:L052601, Nov 2022.

[8] M. Mitrano, A. A. Husain, S. Vig, A. Kogar, M. S. Rak, S. I. Rubeck, J. Schmalian, B. Uchoa, J. Schneeloch, R. Zhong, G. D. Gu, and P. Abbamonte. Anomalous density fluctuations in a strange metal. Proceedings of the National Academy of Sciences, 115(21):5392–5396, 2018.
